# HIV prevalence and access to HIV testing and care in patients with psychosis in South Africa

**DOI:** 10.4102/sajpsychiatry.v29i0.1918

**Published:** 2023-01-31

**Authors:** Mbalenhle P. Mwelase, Vuyokazi Ntlantsana, Andrew Tomita, Bonginkosi Chiliza, Saeeda Paruk

**Affiliations:** 1 Discipline of Psychiatry, College of Health Sciences, University of KwaZulu-Natal, Durban, South Africa; 2 KwaZulu-Natal Research Innovation and Sequencing Platform (KRISP), College of Health Sciences, University of KwaZulu-Natal, Durban, South Africa; 3 Centre for Rural Health, School of Nursing and Public Health, College of Health Sciences, University of KwaZulu-Natal, Durban, South Africa

**Keywords:** psychosis, HIV, South Africa, viral load, prevalence

## Abstract

**Background:**

Human immunodeficiency virus (HIV) and psychosis share a complex bidirectional relationship, with people living with HIV being at increased risk of psychosis and those with psychosis at increased risk of HIV. However, people living with severe mental illness often have limited or reduced access to HIV testing and care.

**Aim:**

This study aimed to determine the prevalence of HIV and describe the access to HIV testing and care among adult patients with recent-onset psychosis who were admitted to a psychiatric hospital in KwaZulu-Natal (KZN) province, South Africa.

**Setting:**

A psychiatric hospital in Pietermaritzburg, KZN province, South Africa.

**Method:**

A retrospective chart review of 294 patients with recent-onset psychosis admitted between May 2018 and November 2020.

**Results:**

A total of 291 (99%) patients had access to HIV testing during the study period, with the HIV seroprevalence rate being 21.5% among the 294 patients; HIV seropositivity was associated with the 25–49 age category (adjusted odds ratio [aOR] = 3.09, 95% confidence interval [CI] 1.27–7.50), female gender (aOR = 9.55, 95% CI 4.40–20.74), current alcohol and cannabis use (aOR = 3.43, 95% CI 1.01–11.62), family history of psychosis (aOR = 3.22, 95% CI 1.03–10.02) and no tertiary education (aOR = 3.7, 95% CI 0.14–0.99). All those living with HIV were on antiretroviral treatment.

**Conclusion:**

This study showed that HIV testing and care was accessible at a psychiatric hospital but the prevalence of HIV in people living with recent onset psychosis remains high.

**Contribution:**

The study findings suggest the importance of integrating mental health and HIV management.

## Introduction

It is estimated that in 2018, there were an estimated 20.6 million (18.2 million – 23.2 million) people living with human immunodeficiency virus (HIV) in Eastern and Southern Africa and 7.52 million in South Africa (SA).^1^ Approximately 85% of these people knew their HIV status in 2018, and an estimated 67% of people living with HIV were on treatment.^2^

First-episode psychosis (FEP) may be defined as the first time someone experiences psychotic symptoms or a psychotic episode and accesses treatment.^3^ Various definitions of psychotic illness by duration of psychosis have been cited in literature, but most studies define recent-onset psychosis as psychotic symptoms with a duration of less than 5 years.^4^

In SA, a study of 63 patients presenting with adult FEP at a psychiatric hospital in Pietermaritzburg, KwaZulu-Natal (KZN) province, found an HIV seroprevalence rate of 23.8%.^5^ In a more recent study, the authors reported a 39.6% prevalence of HIV among all first-presentation psychotic patients attending the emergency department in Gauteng province, SA.^6^ The high prevalence of HIV among people with severe mental illness, such as with psychosis, is further supported by a recent systematic review and meta-analysis on the prevalence of HIV in patients with FEP from Africa. The study reported an HIV prevalence range from 23.8% to 39.6%, while the meta-analysis of their data reported the pooled proportion of HIV in patients living with FEP as 26% (95% confidence interval [CI] 10–42).^7^

As effective antiretroviral treatments (ARTs) are now available, it is critical to diagnose HIV infection early in the mentally ill, especially as they are a high risk population.^8^ In the struggle against HIV and aquired immunodeficiency syndrome (AIDS), the United Nations (UN) and World Health Organization policy documents assert that HIV testing and counselling remain of prime importance.^9^ Furthermore, they estimate that only 10% of people in developing countries have access to voluntary counselling and testing (VCT).^9^

The reported lifetime prevalence of HIV testing among individuals with severe mental illness ranges from 11% to 89%.^10^ A study set in a poor health resource setting of HIV-related admissions at a psychiatric hospital in Bangalore, India, which assessed the profile of 22 549 admissions, found that only 2283 patients (10.12%) were tested for HIV infection, of whom 51 (2.11%) were HIV seropositive.^11^ In a study that surveyed public sector psychiatrists’ attitudes to HIV testing in the Western Cape province, SA, the state-employed psychiatrists were found to not test routinely for HIV infection, mainly because of ethical constraints, with only 14.6% of patients being tested in 2006.^8^ The same study observed a discharge database audit conducted by P. Milligan^12^ at Lentegeur Hospital, Cape Town, SA, over a three year period that found only from 11.9% to 14.6% of patients were tested for HIV.^8^

A more recent cross-sectional study in 10 public sector health facilities in the Ekurhuleni District of SA found that 51.8% of general medical patients reported that they had not received an HIV test in the past 12 months, and 9.6% had ever been offered HIV testing.^13^ A retrospective analysis of the 2008–2010 cycles of the Philadelphia Medical Monitoring Project, United States of America (USA), compared the proportions of HIV-infected adults with and without mental illness. The results showed that the proportion retained in care was similar for individuals with and without mental illness (91.3% vs. 90.3%). However, individuals with mental illness were less likely to be prescribed ART (83.2% vs. 88.7%) and achieve viral suppression (65.9% vs. 74.4%) than those without a mental health diagnosis.^14^ Left untreated, the mental disorders in the HIV-infected person result in a poorer quality of life, interpersonal difficulties, substance use, increased suicide risk and poor adherence to ART.^15^

The literature suggests that while the prevalence of HIV is high in people with mental illness, their access to HIV testing and care may be poor.^8^ This study therefore explored the prevalence of and access to HIV testing and care of patients admitted with recent onset psychosis at a psychiatric hospital in SA.

## Aim

This study aimed to determine the prevalence of HIV and describe the access to HIV testing and care among adult patients with recent onset psychosis who were admitted to a psychiatric hospital in the uMgungundlovu District, KZN province, SA.

## Research method and design

The study entailed a retrospective chart review of all adult patients with recent onset psychosis admitted to a psychiatric hospital.

### Study population and sampling strategy

In this retrospective chart review, all hospital charts of adults with any mental illness aged 18–65 years who were admitted to the psychiatric hospital from 01 May 2018 to 30 November 2020 were identified from the hospital admission register and screened for eligibility criteria by the principal investigator (PI) from the in-patient register. The files which were included were those of patients with recent onset psychosis and who also met the criteria of the Diagnostic and Statistical Manual of Mental Disorders, Fifth Edition (DSM-5)^16^ for schizophrenia spectrum and other psychotic disorders who required antipsychotic medication and admission to a psychiatric hospital. All patients admitted to the hospital were fully assessed by the admitting doctor using a structured assessment format that also included a report on their psychiatric, medical and substance use history from the patient and family, if available. This was verified using the DSM-5 criteria^16^ by the treating psychiatrists during the course of admission.

### Measures

This study utilised a structured sociodemographic and clinical data extraction tool based on the literature review to measure the selected variables. These included: demographic data (age, gender, family history, marital status, occupation and educational level), and clinical data (psychiatric diagnosis, HIV status, comorbid medical disorders, family history of mental illness, substance use history, record of ART and concomitant medication). Data on clinical investigations were also collated, such as HIV enzyme-linked immunosorbent assay (ELISA) rapid test to confirm HIV status, cluster of difference 4 (CD4) count and viral load (VL) (only in PLWHIV). The patients were categorised into three groups based on their HIV status viz. positive, negative or unknown. Access to HIV testing was measured by number of participants who were offered HIV testing with supporting clinical records on file and HIV care in terms of those who received ART if they had HIV infection.

### Data analysis

The data were entered into a Research Electronic Data Capture (REDCap) database (Vanderbilt University, Nashville, Tennessee, United States) and analysed using Stata version 15.1 (StataCorp LLC, College Station, Texas, United States), with descriptive statistics, such as frequencies and percentages, being used to summarise the results. McNemar’s chi-square test was used to test for any association between HIV status, virological suppression and patients’ characteristics, such as age, sex, employment status and family history. Logistic regression models were used to test for the association between HIV status and patient characteristics, with the significance level being set at *p* = 0.05.

### Definitions used in the study

Recent-onset psychosis was psychotic symptom onset within the past five years.^4^ Duration of untreated psychosis (DUP) was measured from the time from manifestation of the first psychotic symptom to initiation of adequate antipsychotic drug treatment.^17^ Viral load was the amount or concentration of a virus in each quantity of blood, saliva, mucus or other bodily fluid, often expressed as the number of viral particles per millilitre of the fluid. Family history of mental illness was defined as positive if first- and second-degree relatives had mental illness.

### Ethical considerations

Ethical approval to conduct the study was obtained from the Biomedical Research Ethics Committee (reference number BREC/00001591/2020) of the University of KwaZulu-Natal (UKZN), the public sector psychiatric hospital and the Department of Health (DOH). The study was conducted in accordance with SA DOH Research Ethics guidelines (2015) and the UKZN policy on research ethics.

### Data storage and management

Data were stored at the university in a password-protected computer with access to researchers only. Data will be stored for five years after the study and then destroyed.

Confidentiality was maintained throughout and after completion of the research. All data had potential identifiers removed and replaced with codes to ensure the anonymity of participants. These codes will be retained separately from the data.

## Results

### Sociodemographic and clinical characteristics

The clinical records of all 294 patients with recent-onset psychosis admitted during the study period were included, and where data were missing, this is indicated accordingly. The sociodemographic and clinical characteristics are stratified by HIV status (Table 1). Most participants admitted were in the 25–49 years age group (63.3%), male (68.7%), unmarried (94.2%) and unemployed (70.1%). The DUP ranged from 0 to 240 months (IQR 1–36 months), and 144 (49.8%) individuals had a DUP greater than 12 months.

**TABLE 1:** Sociodemographic and clinical profile based on known HIV status in people with recent onset psychosis.

Variable	Categories	Total (*n* = 294)		HIV negative (*n* = 227)		HIV positive (*n* = 62)		*p*
		*n*	%	*n*	%	*n*	%	
Age categories (years)	18–24	86	29.3	74	32.6	11	17.7	0.07
	25–49	186	63.3	137	60.4	46	74.2	-
	50 +	22	7.5	16	7.0	5	8.1	-
Gender	Female	92	31.3	53	23.3	37	59.7	< 0.001
	Male	202	68.7	174	76.7	25	40.3	
Marital status	Not married	277	94.2	213	93.8	60	96.8	0.37
	Married, widowed or divorced	17	5.8	14	6.2	2	3.2	-
Educational level	Less than tertiary (Grade 12 or less)	243	82.7	185	81.5	54	87.1	0.30
	Tertiary	51	17.3	42	18.5	8	12.9	-
Employment status	Employed	50	17.0	41	18.1	9	14.5	0.01
	Unemployed	206	70.1	164	72.2	38	61.3	-
	Disability grant	38	12.9	22	9.7	15	24.2	-
Current substance use	No alcohol or cannabis	137	46.6	107	47.1	29	46.8	0.96
	Alcohol and/or cannabis	157	53.4	120	52.9	33	53.2	-
Family history	Psychosis	24	8.2	16	7.0	7	11.3	0.06
	Nonpsychotic mental illness	47	16.0	42	18.5	5	8.1	-
	Substance use disorder	18	6.1	11	4.8	7	11.3	-
	None	205	69.7	158	69.6	43	69.4	-
Duration of untreated psychosis (months)	0–11	145	50.2	109	49.1	33	53.2	0.57
	12 +	144	49.8	113	50.9	29	46.8	-
HIV status	Negative	227	78.5	-	-	-	-	-
	Positive	62	21.5	-	-	-	-	-
	Unknown	5	0.01	-	-	-	-	-

### HIV prevalence

The HIV seroprevalence rate was 21.5% (*n* = 62), with women accounting for 59.7% (*n* = 37), having a significantly higher HIV prevalence than the men (*p* ≤ 0.001), all of whom received ART while admitted.

### Access to HIV testing and care

Almost all patients (291 of the 294) accessed HIV testing (or were offered it) whilst admitted to a psychiatric hospital. Five (1.7%) of the 294 patients did not have an HIV test result, three (1%) of whom had no documentation of whether HIV test was offered or the result, one (0.3%) of whom had refused to test and another (0.3%) of whom had tested but there was no record of the result.

## Association between HIV status and patient characteristics

The association between HIV seropositivity and patient characteristics are presented in Table 2. The odds of being HIV positive were significantly higher for patients in the 25–49 age category (adjusted odds ratio [aOR] = 3.09, 95% CI 1.27–7.50), women (aOR = 9.55, 95% CI 4.40–20.74) and those who self-reported current alcohol and cannabis use (aOR = 3.43, 95% CI 1.01–11.62). A family history of psychosis (aOR = 3.22, 95% CI 1.03–10.02) also significantly increased the odds of living with HIV, while the odds of HIV were 63% lower for those who had a tertiary education compared with those who did not. There was no difference in the duration of untreated psychosis between the HIV-negative and -positive groups.

**TABLE 2:** Association between HIV status and sociodemographic and clinical characteristics in people with recent-onset psychosis.

Variable	Categories	Overall		
		aOR	s.e.	95% CI
Age categories†	[18–24]			
	25–49	3.09	1.40	1.27–7.50
	50 +	1.34	0.98	0.32–5.63
Sex‡	[Male]	-	-	-
	Female	9.55	3.78	4.40–20.74
Marital status§	[Not married]	-	-	-
	Married, widowed or divorced	0.27	0.23	0.05–1.39
Educational level¶	[Less than tertiary]	-	-	-
	Tertiary	0.37	0.19	0.14–0.99
Employment status††	[Employed]	-	-	-
	Unemployed	0.90	0.42	0.36–2.24
	Disability grant	2.46	1.49	0.75–8.06
Current substance use ‡‡	[No alcohol or cannabis]	-	-	-
	Alcohol or cannabis use	2.58	0.99	1.22–5.48
Family history§§	[None]	-	-	-
	Psychosis	3.22	1.86	1.03–10.02
	Nonpsychotic mental illness	0.49	0.27	0.16–1.46
	Substance use disorder	3.43	2.13	1.01–11.62
Duration of untreated psychosis (months)¶¶	[0–11]	-	-	-
	12 +	0.73	0.25	0.37–1.42

aOR, adjusted odds ratio; s.e., standard error; CI, confidence interval.

† , comparison group is 18–24;

‡ , comparison group is male;

§ , comparison group is not married;

¶ , comparison group is less than tertiary;

†† , comparison group is employed;

‡‡ , comparison group is no alcohol or cannabis use;

§§ , comparison group is no family history of psychosis;

¶¶ , comparison group is 0–11.

## Viral load and patient characteristics

Of the 62 PLWHIV and recent onset psychosis, 37 (66.07%) were virologically suppressed, as indicated by a VL of lower than detectable, and 19 (33.93%) were virologically unsuppressed. The study was not able to establish from the records if individuals were newly diagnosed with HIV or were re-initiated on ART after defaulting treatment. The associations between VL measure and patients’ sociodemographic and clinical characteristics are presented in Table 3, with being male and having a lower CD4 count being significantly associated with being virologically unsuppressed (*p* ≤ 0.01).

**TABLE 3:** Association between viral load and sociodemographic and clinical characteristics in people with recent onset psychosis and HIV.

Variable	Categories	VL suppressed (*n* = 37)		VL unsuppressed (*n* = 19)		*p*
		*n*	%	*n*	%	
Age categories	18–24	6	60.0	4	40.0	0.29
	25–49	26	63.4	15	36.6	-
	50 +	5	100.0	0	0.0	-
Sex	Female	28	80.0	7	20.0	< 0.01
	Male	9	42.9	12	57.1	-
Marital status	Not married	35	64.8	19	35.2	0.43
	Married, widowed or divorced	2	100.0	0	0.0	-
Educational level	Less than tertiary	32	65.3	17	34.7	0.56
	Tertiary	5	71.4	2	28.6	-
Employment status	Employed	6	85.7	1	14.3	0.42
	Unemployed	21	60.0	14	40.0	-
	Disability grant	10	71.4	4	28.6	-
Current substance use	No alcohol or cannabis	17	63.0	10	37.0	0.64
	Alcohol or cannabis use	20	69.0	9	31.0	-
Family history	Psychosis	4	80.0	1	20.0	0.34
	Nonpsychotic mental illness	5	100.0	0	0.0	-
	Substance use disorder	4	57.1	3	42.9	-
	None	24	61.5	15	38.5	-
Duration of untreated psychosis (months)	0–11	20	66.7	10	33.3	0.92
	12 +	17	65.4	9	34.6	-
CD4 count	-	745 (median)	-	416 (median)	323 556 (IQR)	< 0.01

VL, viral load; IQR, interquartile range.

## Discussion

In this study, the main findings were that 21.5% of patients admitted over the study period with recent-onset psychosis were living with HIV; its seroprevalence was associated with age 25–49 years, female gender, lower educational level, family history of psychosis and current alcohol or cannabis use. There was a very high rate of HIV testing (98%) and care, as all PLWHIV received ART while admitted. Nineteen of the 37 (51.3%) patients with HIV were virologically unsuppressed, with men being less likely to be virally suppressed.

The 21.5% prevalence rate of HIV among patients admitted with recent onset psychosis is slightly higher than the South African general population estimate of 19.0% HIV prevalence in adults aged 15–49 years.^1^ The prevalence in this study is also lower than the estimated 26% rate in the recent systematic review of HIV prevalence in FEP in Africa.^7^ Hopefully, the results of this study suggest that HIV prevalence is decreasing in those who are mentally ill and moving towards the general population rates, but this will need to be explored further in larger prospective studies.

In this study, only five patients (1.7%) did not have an HIV test result, which indicates that access to HIV testing was good, and an extremely small proportion were missed or had an unknown HIV status; however, clinicians should endeavour to offer all patients HIV testing and care and document outcomes. This is in stark contrast to earlier studies, which found poor or inadequate access to HIV testing for those with mental illness.^8^ The reported lifetime prevalence of HIV testing among individuals with severe mental illness ranges from 11% to 89%, indicating wide variability in HIV testing in this group.^10^ In a retrospective chart review study in Johannesburg, SA, the clinical features, inpatient care and accessibility to ART of mental healthcare users who were admitted to an acute psychiatric unit found that only 17.4% were tested for HIV. The low rates of testing for HIV status were because of ethical considerations about obtaining consent.^18^ The reasons for inadequate HIV testing in previous studies may be multifactorial and include concerns on lack of informed consent, stigma and bias.^19^ The right of patients to consent or refuse consent to medical treatments is an important right that has developed out of the ethical concepts of freedom, rights and autonomy. It is incorporated into all legal frameworks in most countries around the world and certainly in all Western-style democracies. There are, however, important exceptions to this rule that have ethical, legal and practical reasons. It is acknowledged that some persons do not have the mental capacity to make an informed choice, in which case it is ethical to consider acting in their best interest.^20^ A study in a Cape Town, SA, examined the relationship between HIV testing history, attitudes towards testing and AIDS stigma and found that compared with people who had been tested, individuals who were not tested demonstrated significantly greater AIDS-related stigmas, ascribing greater shame, guilt and social disapproval to people living with HIV.^21^ As HIV and ART are less stigmatised and health policies and guidelines now encourage routine testing and treatment, there may be improved levels of HIV testing and care in mental health services. This study suggests that the current Department of Health guidelines^22^ to test all patients at any facility are proving effective, and some clinical facilities may have embraced HIV testing for the mentally ill.

In this study, the HIV risk in people with recent-onset psychosis was associated with the age group 25–49 years, which is also consistent with HIV prevalence rates in the general population in KZN, SA, among people aged 15–49 in 2017, which was at 27%.^23^ This suggests that young adults remain very vulnerable, and HIV prevention programmes, including advocacy initiatives, should target this group in those with severe mental illness.

In this study, women contributed 59.7% of the HIV-positive group and had a significantly higher HIV rate than the men (*p* ≤0.001), which is consistent with the literature.^18,24,25,26,27^ Studies suggest that there is a female preponderance, often in 20–40 year age range, presenting with HIV and psychosis.^18^ Another study examined the HIV seroprevalence among psychiatric patients admitted to a public psychiatric institution in KZN between July and November 2003 by conducting anonymous HIV testing among 151 patients and found that 40 (26.5%) were positive, and that women were more likely to be infected than men (aOR = 2.74; 95% CI = 1.25–6.04; *p* = 0.012).^24^ In another chart review of patients with psychosis admitted to a psychiatric hospital in Durban, SA, PLWHIV and psychotic disorders were also more likely to be female (74.0%),^25^ the literature suggesting that they are the more vulnerable to contracting HIV, with prevention strategies needing to target them in particular.

The HIV prevalence was also higher in those without a tertiary education compared with those with, which is supported by other South African studies in people with psychosis.^28^ It is also important to be cognisant that there has been mixed evidence in studies conducted in sub-Saharan Africa on this subject. In a systematic review that assessed the evidence on the association between educational attainment and risk of HIV infection, it was observed that this relationship may be changing over time in sub-Saharan Africa. It was found that studies conducted before 1996 tended to find no association with education level and HIV-infection risk among the more educated, while a larger proportion of those studies conducted thereafter identified a lower risk of HIV among the more educated.^29^ This reinforces the need to ensure HIV-prevention measures reach all strata of society and include current approaches that focus primarily on providing information, distributing condoms and treating sexually transmitted infections, with a focus on increasing HIV education in vulnerable populations.^29^

Individuals who report current substance use were more than three times more likely to have HIV in this study; this is consistent with international literature, which reports that substance use also increases the risk of HIV transmission, as it may contribute to increased risky behaviour.^30,31^ Local studies of HIV seropositivity in the mentally ill in KZN have, however, not shown an increase in substance use in PLWHIV and psychosis;^5,25^ possibly these studies are limited by sample size and methodology. This finding needs further investigation but does highlight the need for a more comprehensive assessment and holistic care package that encompasses care for medical conditions, such as HIV, while managing comorbid mental illness and substance use.

In this study of those living with HIV, 37 (66.07%) were virologically suppressed; this rate is lower than that reported in the international literature for PLWHIV but higher than that in a recent South African general population.^32,33^ A study that assessed the patterns of viral suppression among patients living with HIV across the USA showed an increasing rate of viral suppression, from 32% in 1997 to 86% in 2015.^32^ A South African cross-sectional survey conducted among 15–49-year old pregnant women attending antenatal care at 1595 nationally representative public facilities found that of 10 052 HIV-positive participants with VL data, 56.2% were virally suppressed.^33^

Nineteen (33.93%) of the participants in our study were virologically unsuppressed, which is concerning, as it suggests that the HIV care of patients with psychosis needs to be intensified. These participants were either newly diagnosed with HIV or were re-initiated on ART after defaulting treatment. However, this was difficult to determine from the records available, as anecdotal evidence suggests that patients may not disclose if diagnosed with HIV previously. This suggests a need to consider more active follow-up of those with psychosis and HIV in terms of adherence support to improve outcomes.

### Study limitations

This was a single public sector hospital–based study, which may introduce sample bias and limit generalisability to community samples. However, it does provide a sample representing the clinical profile of patients with recent onset psychosis and the access to HIV testing and prevalence of HIV in a resource-constrained setting that drains large urban and rural populations. Other limitations include that the cross-sectional nature of the study design means that changes over time may be missed. The study was also not able to establish from the records if individuals were newly diagnosed with HIV or were re-initiated on ART after defaulting treatment. In addition, the retrospective chart review means that the data may be limited by the quality of record-keeping and depend on clinician-assigned diagnoses, which are less reliable than structured tools from the clinical records. However, all patients were assessed by a psychiatrist during the admission to review their diagnosis, and the PI also reviewed the clinical notes and diagnoses based on DSM-5 diagnostic criteria.^16^

## Conclusion

This study found that while HIV testing and care were available, the prevalence of HIV in people living with recent onset psychosis remains high and that almost a third were not virologically suppressed. This suggests the importance of further interventions to integrate mental health and HIV prevention and promotion, treatment and rehabilitation, with a particular focus on those vulnerable to the comorbidity (women, those with lower education and young adults). This will hopefully improve mental and physical health outcomes. Further longitudinal studies with interventions that seek to screen for the dual burden of disease (psychosis and HIV) and provide integrated care with a multidisciplinary team are required.
